# Chicago Veterinary College

**Published:** 1895-09

**Authors:** 


### CHICAGO VETERINARY COLLEGE.

The session of 1895–96 will commence on October 3, and extend to the last week in March, a course of six months, for two years, inclusive of examinations. We note no change in the matriculation examination nor requirements for graduation. Several changes and additions are noted in the faculty for the ensuing year.

Dr. F. S. Billings, professor of general pathology and microscopic anatomy, is succeeded by Arthur P. Edwards, M.D., instructor in medicine and physical diagnosis, Northwestern University Medical School.

Dr. A. H. Baker relinquishes the chair of cattle pathology and is succeeded by M. R. Trumbower, D.V.S., State veterinarian of Illinois, who will also instruct in the subject of meat and milk inspection.

A. McBride, M.D.C., assistant to the chair of theory and practice, has been succeeded by P. L. Darcey, M.D.C., Class of 1894.

We note the title of M.D.C. after the names of Prof. A. H. Baker and F. S. Billings, neither of whose names appear in the list of graduates, the former having received his veterinary degree at the Montreal Veterinary College. In the announcement of 1894–95 the letters M.D.C. appear after the name C. E. Sayre, professor of dental surgery, while the announcement of 1895–96 designates the letters D.V.S. Dr. Sayre is a graduate of the Chicago Veterinary College, class of 1887. J. F. Ryan, professor of helminthology, is noted by the letters M.D.C., though he does not appear as a graduate of the Chicago Veterinary College. We were under the impression that he also was a graduate of Montreal, class of 1877. This confusion of titles makes one wish that there was a single uniform degree for veterinary graduates in North America.

---

